# Spatial characteristics and the epidemiology of human infections with avian influenza A(H7N9) virus in five waves from 2013 to 2017 in Zhejiang Province, China

**DOI:** 10.1371/journal.pone.0180763

**Published:** 2017-07-27

**Authors:** Haocheng Wu, XinYi Wang, Ming Xue, Melanie Xue, Chen Wu, Qinbao Lu, Zheyuan Ding, Xiaoping Xv, Junfen Lin

**Affiliations:** 1 Zhejiang Province Center for Disease Control and Prevention, Hangzhou, Zhejiang Province, China; 2 Key Laboratory for Vaccine, Prevention and Control of Infectious Disease of Zhejiang Province, Hangzhou, Zhejiang Province, China; 3 Hangzhou Centre for Disease Control and Prevention, Hangzhou, Zhejiang, Province, China; 4 Kingston University UK, London, United Kingdom; Icahn School of Medicine at Mount Sinai, UNITED STATES

## Abstract

**Background:**

The five-wave epidemic of H7N9 in China emerged in the second half of 2016. This study aimed to compare the epidemiological characteristics among the five waves, estimating the possible infected cases and inferring the extent of the possible epidemic in the areas that have not reported cases before.

**Methods:**

The data for the H7N9 cases from Zhejiang Province between 2013 and 2017 was obtained from the China Information Network System of Disease Prevention and Control. The start date of each wave was 16 March 2013, 1 July 2013, 1 July 2014, 1 July 2015 and 1 July 2016. The *F* test or Pearson’s chi-square test were used to compare the characteristics of the five waves. Global and local autocorrelation analysis was carried out to identify spatial autocorrelations. Ordinary kriging interpolation was analyzed to estimate the number of human infections with H7N9 virus and to infer the extent of infections in the areas with no cases reported before.

**Result:**

There were 45, 94, 45, 34 and 80 cases identified from the first wave to the fifth, respectively. The death rate was significantly different among the five waves of epidemics (*χ*^2^ = 10.784, *P* = 0.029). The age distribution (*F* = 0.903, *P* = 0.462), gender (*χ*^2^ = 2.674, *P* = 0.614) and occupation(*χ*^2^ = 19.764, *P* = 0.407) were similar in each period. Most of the cases were males and farmers. A significant trend (*χ*^2^ = 70.328, *P*<0.001) was identified that showed a growing proportion of rural cases. There were 31 high-high clusters and 3 high-low clusters at the county level among the five waves and 12, 8, 2, 9 and 3 clusters in each wave, respectively. The total cases infected with the H7N9 virus were far more than those that have been reported now, and the affected areas continue to expand. The epidemic in the north of Zhejiang Province persisted in all five waves. Since the second wave, the virus spread to the south areas and central areas. There was an obvious decline in the infected cases in the urban areas, and the epidemics mostly occurred in the rural areas after the fourth wave. The epidemic was relatively dispersed since the third wave had fewer than two cases in most of the areas and showed a reinforcing trend again in the fifth wave.

**Conclusions:**

The study revealed that there were few differences in the epidemiologic characteristics among the five waves of the epidemic. However, the areas where the possible epidemic circulated was larger than reported. The epidemic cross-regional expansion continued and mostly occurred in rural areas. Continuous closure of the live poultry market (LPM) is strongly recommended in both rural and urban areas. Illegal and scattered live poultry trading, especially in rural areas, must be forbidden. It is suggested too that a more rigorous management be performed on live poultry trade and wholesale across the area. Health education, surveillance of cases and pathogenicity should also be strengthened.

## Introduction

The H7N9 virus is a virus that reasserts multiple times and whose gene fragments are obtained from H7N9, H9N2 and H7N3 subtypes of the influenza A virus[1–3]. The first case of human infections with the H7N9 virus occurred in the spring of 2013 in Eastern China[4,5]. The epidemic caused great concern due to the increasing number of cases, the expansion of the affected areas, the high fatality rate, mutation of the virus and the stigma attached to the virus [6,7]. To date, five waves of H7N9 epidemics have emerged in mainland China. There were 775 laboratory-confirmed infections of A(H7N9) virus across 16 provinces and 3 municipalities as of August 31, 2016[8]. In addition, 23 travelers infected with A(H7N9) exported the virus to Hong Kong (sixteen cases), Taiwan (four cases), Canada (two cases), and Malaysia (one case), leading to four deaths [8]. The fifth wave of the epidemic occurred in autumn 2016, and incidences far exceeded those in the corresponding period of 2015. It should be noted that Zhejiang is a province located in the Yangtze River Delta region of southeastern China that is well recognized as the original source of the H7N9 outbreaks [9]. This province was the most seriously affected area, accounting for nearly 30% of total cases reported and 25% of the total fatal cases during the last four waves in mainland China[10].

There are likely to be many more cases than those reported due to there being an unknown number of mild and subclinical infections [11]. The objectives of this study are to identify the epidemiological characteristics and the distribution of H7N9 virus in human infection from 2013 to 2017. The Kriging spatial interpolation methods will be applied to estimate the number of human infections with H7N9 virus and to infer the extent of infections in areas that currently have no reported cases.

## Methods

### Definition of the five waves

Based on the date of onset, the first wave of H7N9 virus circulation occurred from 13 March to 30 June 2013. In the first wave, the first and last cases occurred on 13 March and 18 April 2013, respectively. The second wave occurred from 1 July 2013 to 30 June 2014, with the first and last cases occurring on 7 October 2013 and 3 June 2014, respectively. The third wave occurred from 1 July 2014 to 30 June 2015. In the third wave, the first and last cases occurred on 17 November 2014 and 28 May 2015, respectively. The fourth wave occurred from 1 July 2015 to 30 June 2016. In the fourth wave, the first and last cases occurred on 18 September 2015 and 24 June 2016, respectively. The fifth wave occurred from 1 July 2016 to 31 March 2017, with the first case occurring on 28 September 2016.

### Patient/cluster definition and data collection

#### The patient definition

The diagnosis of the infections confirmed with the H7N9 virus was based on the Chinese Guideline of Diagnosis and Treatment for Human Infections with the Avian Influenza A(H7N9) Virus issued by the National Health and Family Planning Commission of the People’s Republic of China[7,12,13]. The definition of the confirmed case is clinical manifestation with acute influenza (fever, cough, coryza, difficulty breathing) or a history of contact with a confirmed or suspected case and a laboratory test that includes subtype confirmation by several parameters. These include PCR, viral isolation or no less than a four-fold increase in virus-specific serum antibodies isolated from paired positive sera samples [3,12,13].

#### The cluster definition

According to the Chinese Guideline of Epidemic Prevention and Control for Human Infections with the Avian Influenza A(H7N9) Virus issued by the National Health and Family Planning Commission of the People’s Republic of China[14], the definition of the cluster is that two confirmed cases emerged within 7 days and in small areas, such as one family or one community.

#### The data collection

The confirmed case must be reported through the China Information Network System of Disease Prevention and Control by medical staff. The data of H7N9 cases in Zhejiang Province from 2013 to 2017 were obtained from this network system(S1 File).

#### Profile of Zhejiang Province

**Fig 1:**
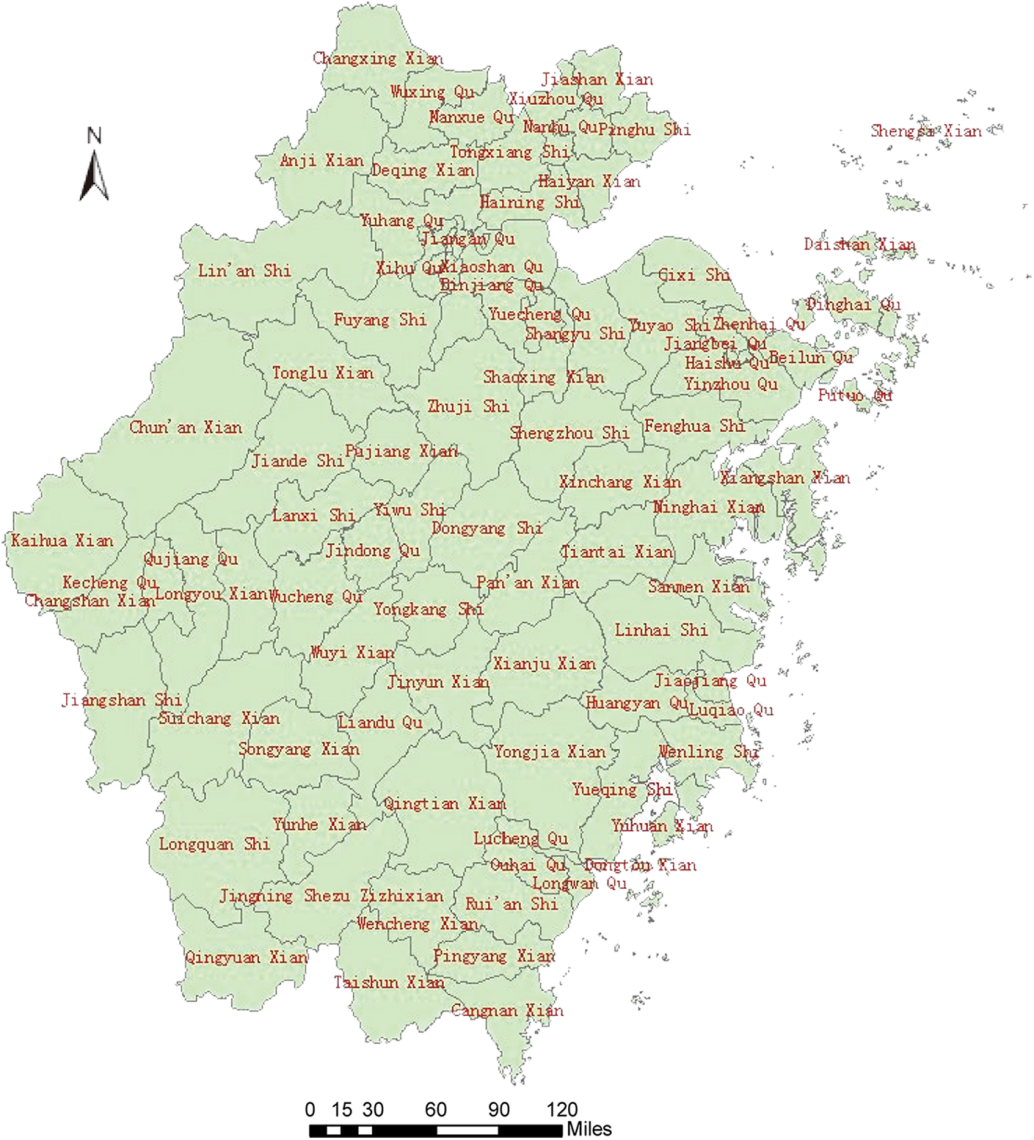
Maps of Zhejiang Province, China with area name.

The Zhejiang Province is in the southeast China between longitudes 118^o^E-123^o^E and latitudes 27^o^N-32^o^N. There are two sub-provincial cities (HangZhou and Ningbo) and nine prefecture-level cities, including Wenzhou, Huzhou, Jiaxing, Shaoxing, Jinhua, Zhoushan, Quzhou, Taizhou and Lishui, which cover 90 counties(Fig 1) in Zhejiang Province.

### Spatial autocorrelation analysis

A global and local autocorrelation analysis using the Moran’s I was carried out to identify spatial autocorrelations. According to the theory, the closer two locations were to each other, the more likely for the incidence rates have an impact on each other [15]. The global autocorrelation demonstrates the spatial distribution clusters over areas [16]. The Local Moran’s Index was used to classify the autocorrelations into positive and negative values. When there were similar high values or low values of the incidence rates, they were defined as having a positive autocorrelation (represented as High-High or Low-Low autocorrelation). If the attributes held opposing high and low values, they were considered to have a negative autocorrelation (represent as High-Low or Low-High autocorrelation) [17].

### Ordinary kriging interpolation

Ordinary kriging (OK) is one of the most commonly used kriging interpolation methods. It has been proven to be the best linear unbiased estimate for variables in regions where the data are spatially autocorrelative[18]. The unsampled value is computed as a linear combination of neighboring observations[19,20]:

$$z^{*}(x_{0},y_{0})=\sum_{i=1}^{n}\lambda_{i}Z_{obs}(x_{i},y_{i})$$

where z^*^(x_0_,y_0_) is the predicted value that is spatially located at point (x_0_,y_0_), λ_i_ is the weight associated with the measured value of Z at the location (x_i_,y_i_), and the weights are derived from the kriging equation using a semivariance function. The parameters of the semivariance function and the nugget effect can be estimated by an empirical semivariance function[19]:

$$\gamma (h)=\frac{1}{2N(h)}\sum_{i=1}^{N(h)}{\left[Z_{obs}(x_{i}+h_{x},y_{i}+h_{y})\;\text{-}\;Z_{obs}(x_{i},y_{i})\right]}^{2}$$

where γ(h) is the semivariance value at the distance interval h, N(h) is the number of sample pairs within the distance interval h, and Z_obs_(x_i_ + h_x_,y_i_ + h_y_) Z_obs_(x_i_,y_i_) are sample values at two points separated by the distance interval h.

### Statistical analysis

The *F* test was employed to compare the mean age of H7N9 cases between the five waves. Pearson’s chi-square or Fisher’s exact test were used to compare the occupational and sex distribution of the cases between the five waves. Pearson’s chi-square test and the chi-square test for trend were used to compare the death rate and the proportion of rural cases between the five waves. The Bonferroni *t*-test was employed to further compare the two indexes between each wave. The methods above were computed by SAS9.2 (SAS Institute Inc., Cary, NC). ArcGIS software (version 10.1, SERI Inc.; Redlands, CA, USA) was used for the spatial autocorrelation analysis and the Ordinary kriging interpolation. The county was adopted as the geographic unit to calculate the spatial autocorrelation. A *P* value of less than 0.005 was considered to be statistically significant for the Bonferroni *t*-test and a *P* value of less than 0.05 for the other tests.

### Ethical review

This study was reviewed and approved by the Ethics Committee of the Zhejiang Provincial Center for Disease Control and Prevention. All the data for the individuals were kept confidential as requested.

## Results

### Epidemiologic characteristics in five waves

From March 2013 to March 31, 2017, there were a total of 298 H7N9 human infections and 112 fatal cases reported in the Zhejiang Province, China. There were 45, 94, 45, 34 and 80 cases identified from the first wave to the fifth epidemic, respectively. In each wave, 10, 39, 24, 13, and 26 fatalities were found, respectively, with significant differences among them (*χ*^2^ = 10.784, *P* = 0.029). There was no significant difference among the latest four waves (*χ*^2^ = 2.662, *P* = 0.447), and the significant difference at the 0.005 level was only identified between the first and the third epidemic (*χ*^2^ = 9.265, *P* = 0.002). The highest death rate was in the third wave (53.3%) and the lowest was in the first (22.2%). However, there were no significant trend in the death rate among the five waves (*χ*^2^ = 0.034, *P* = 0.854).

There was a total of 144 rural cases reported, with significant differences in the proportion among the five waves (*χ*^2^ = 73.361, *P*<0.001). In total, 10, 24, 21, 24 and 65 rural cases were found in each wave, with a significant trend (*χ*^2^ = 70.328, *P*<0.001) identified showing a growing proportion of rural cases. The proportion in the fifth wave was significantly different from that in the first, the second and the third wave (*χ*^2^ = 41.811, 53.697, 16.047, *P*<0.001, *P*<0.001, *P*<0.001, respectively), but similar to the fourth wave (*χ*^2^ = 1.584, *P* = 0.208). All the above results were shown in Table 1.

**Table 1:** Characteristics of H7N9 human infections by waves in Zhejiang Province, China from March 2013- January 2017.

Characteristics	Total(n = 298)	Wave						
		First(n = 45)	Second(n = 94)	Third(n = 45)	Fourth(n = 34)	Fifth(n = 80)	*F*/*χ*^2^	*P* value
Death,n(%)	112(37.6)	10(22.2)	39(41.5)	24(53.3)	13(38.2)	26(32.5)	10.784	0.029
Male,n(%)	188 (63.1)	28(62.2)	64(68.1)	30(66.7)	19(55.9)	47(58.8)	2.674	0.614
Rural areas,n (%,versus urban areas)	144(48.3)	10(22.2)	24(25.5)	21(46.7)	24(70.6)	65(81.3)	73.361	<0.001
Age(years)	57.2±0.88	59.7±2.15	55.3±1.81	55.9±1.97	58.9±2.41	58.0±1.59	0.903	0.462
Occupation,n(%)							19.764	0.407
Farmer	135(45.3)	16(35.6)	41(43.6)	21(46.7)	15(44.1)	42(52.5)		
Retiree	59(19.8)	14(31.1)	18(19.1)	9(20.0)	7(20.6)	11(13.8)		
Worker	35(11.7)	3(6.7)	12(12.8)	5(11.1)	6(17.6)	9(11.3)		
Homemaker	23(7.7)	4(8.9)	3(3.2)	6(13.3)	2(5.9)	8(10.0)		
Child	3(1.0)	0(0.0)	3(3.2)	0(0.0)	0(0.0)	0(0.0)		
Other	43(14.4)	8(17.8)	17(18.1)	4(8.9)	4(11.8)	10(12.5)		

**Fig 2:**
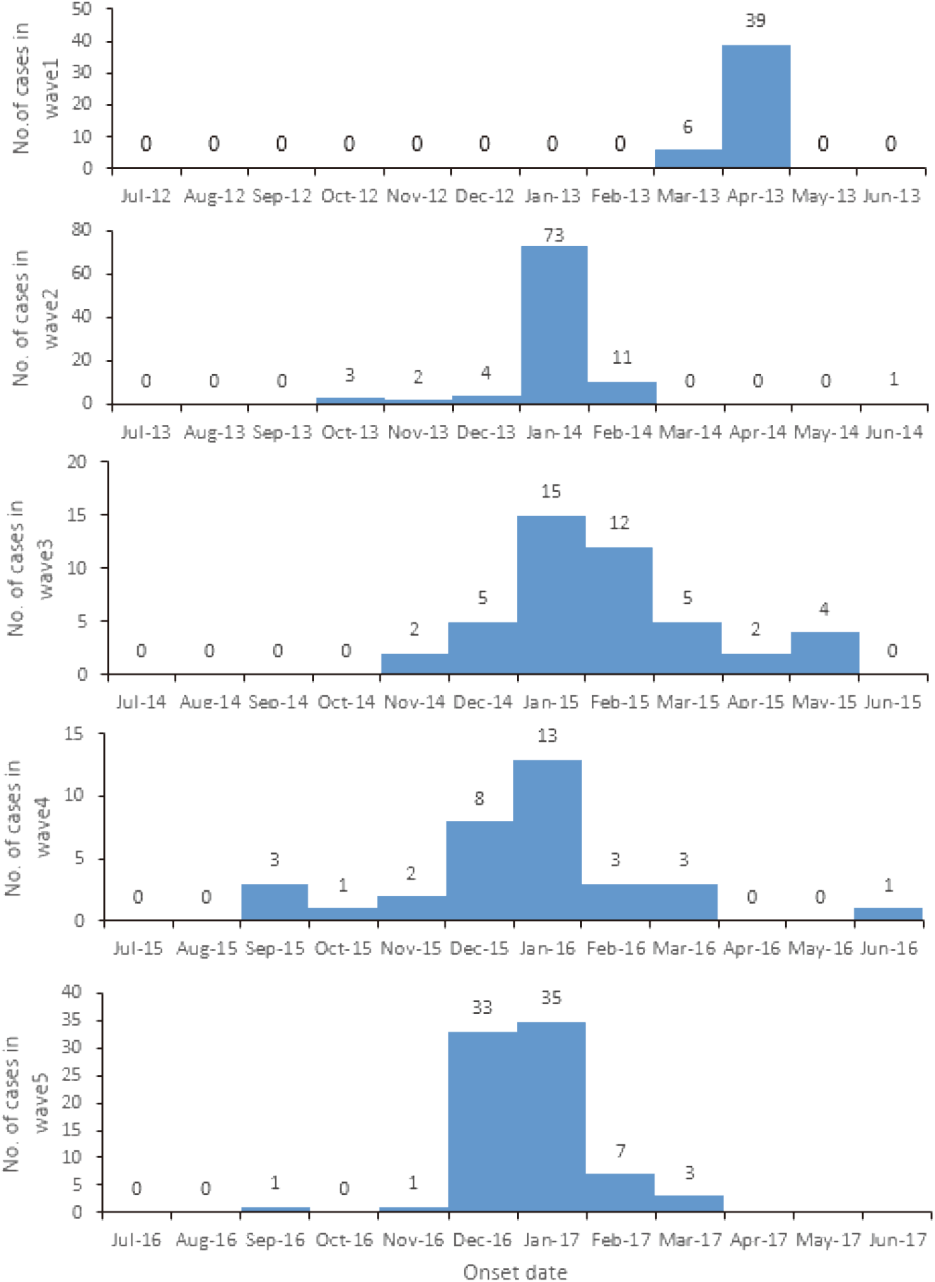
The epidemic curve of H7N9 human infections in Zhejiang Province, China, March 2013-Janurary 2017 at monthly interval.

An obvious seasonality was present for the occurrence of H7N9 human infection across the five waves (Fig 2). Cases emerged in March 2013 and grew rapidly, with a peak in April 2013, and then declined. No case was reported from May to September until a new case remerged in October 2013. It should be noted that most cases occurred in January in the following three waves, accounting for an average of 58.4% of the total cases in each wave. The peak incidence of the fifth wave occurred in December 2016, which was earlier than the other waves, and continued to January, 2017, followed by a sharp decline in February and March (7 and 3 cases, respectively). A few cases (9 cases, 4.13% of total) were identified during May and September in the previous four waves. Of the total cases, male cases predominated, accounting for an average of 63.1% in the five waves. Gender distribution did not differ significantly among the five waves (*χ*^2^ = 2.674, *P* = 0.614). The mean age of the cases was 57.2±0.88 in the five waves. For each wave it was 59.7±2.15,55.3±1.81,55.9±1.97,58.9±2.41,58.0±1.59, respectively, with no significant difference (*F* = 0.903, *P* = 0.462) among them. No significant difference was found among the five waves in occupation (*χ*^2^ = 19.764, *P* = 0.407), with farmers making up most of the cases (45.3% average). However, there was a greater proportion of farmers in the fifth wave (52.5%) than in the previous waves. Few cases in children were reported to date in Zhejiang Province, China (1.0% average).

### Spatial clustering distribution

According to the global autocorrelation analysis (Table 2), the distribution of the cases infected with the H7N9 virus in the first, second and fourth waves presented a spatial autocorrelation. The Moran's I Index was highest in the first wave (Index = 0.8161, *P* value<0.001), followed by an index of 0.2658 in the second wave and 0.1468 in the fourth one. Spatial autocorrelation was not found in the third and fifth waves among the whole province.

**Table 2:** The global spatial autocorrelation of H7N9 human infections in Zhejiang Province among five waves.

Wave	Moran’s I Index	Moran’s I Z-score	Moran’s I *P*-value
The first	0.8161	12.3646	<0.001
The second	0.2658	4.0801	<0.001
The third	0.0649	1.0644	0.2871
The fourth	0.1468	2.2533	0.0242
The fifth	0.0173	0.4000	0.6891

Based on the result of the local Moran's I autocorrelation (Table 3), there were 31 high-high clusters and 3 high-low clusters in total at the county level through the five waves, with 12, 8, 2, 9 and 3 clusters in each wave, respectively. It should be noted that Anji county, Deqing county, Yuhang county, Xiaoshan county, Shangcheng county, Shaoxing county and Yuecheng county had long-term high-high clusters throughout the five waves (4,3,3,3,2,2,2 high-high clusters respectively).

**Table 3:** The local spatial autocorrelation of H7N9 human infections in Zhejiang Province among five waves.

Waves	Area	LMi Index	LMi Z score	LMi *P*-value	Correlation Type	Number of cases
The first	Deqing	0.0007	7.1125	<0.001	High-High Cluster	2
The first	Anji	0.0001	2.5485	0.0100	High-High Cluster	2
The first	Changxing	0.0001	2.8303	<0.005	High-High Cluster	2
The first	Xiacheng	0.0041	13.2405	<0.001	High-High Cluster	4
The first	Shangcheng	0.0054	15.932	<0.001	High-High Cluster	8
The first	Gongshu	0.0007	2.4489	0.0100	High-High Cluster	1
The first	Wuxing	0.0004	5.3071	<0.001	High-High Cluster	3
The first	Nanxun	0.0003	3.6801	<0.001	High-High Cluster	3
The first	Xiaoshan	0.0023	15.7486	<0.001	High-High Cluster	5
The first	Yuhang	0.0009	6.7498	<0.001	High-High Cluster	2
The first	Jianggan	0.0036	13.0148	<0.001	High-High Cluster	5
The first	Xihu	0.0022	7.4132	<0.001	High-High Cluster	3
The second	Shaoxing	0.001	7.4647	<0.001	High-High Cluster	4
The second	Deqing	0.0004	3.7494	0.0002	High-High Cluster	5
The second	Anji	0.0001	3.488	0.0005	High-High Cluster	3
The second	Shangcheng	0.0007	1.974	0.0484	High-High Cluster	3
The second	Binjiang	0.0008	2.7357	0.0062	High-High Cluster	3
The second	Yuecheng	0.0014	9.9447	<0.001	High-High Cluster	6
The second	Xiaoshan	0.0018	12.2282	<0.001	High-High Cluster	11
The second	Yuhang	0.0008	5.9461	<0.001	High-High Cluster	6
The third	Jindo.ng	0.0002	2.2784	0.0227	High-High Cluster	2
The third	Xianju	-0.0001	-2.5061	0.0122	High-Low Cluster	2
The fourth	Shaoxing	0.0004	2.657	0.0079	High-High Cluster	1
The fourth	Haining	0.0004	4.1655	<0.001	High-High Cluster	3
The fourth	Lin'An	0.0004	3.8097	<0.001	High-High Cluster	2
The fourth	Deqing	0.0001	2.7589	<0.0058	High-High Cluster	2
The fourth	Anji	0.0003	6.6422	<0.001	High-High Cluster	1
The fourth	Yuecheng	0.0005	3.8667	<0.001	High-High Cluster	2
The fourth	Xiaoshan	0.0013	8.8283	<0.001	High-High Cluster	4
The fourth	Yinzhou	-0.0004	-2.5789	0.0099	High-Low Cluster	2
The fourth	Yuhang	0.0007	4.6961	<0.001	High-High Cluster	2
The fifth	Wencheng	0.0002	2.9295	0.0034	High-High Cluster	3
The fifth	Yueqing	-0.0002	-2.4287	0.0152	High-Low Cluster	3
The fifth	Anji	0.0003	7.9654	<0.001	High-High Cluster	4

**Fig 3:**
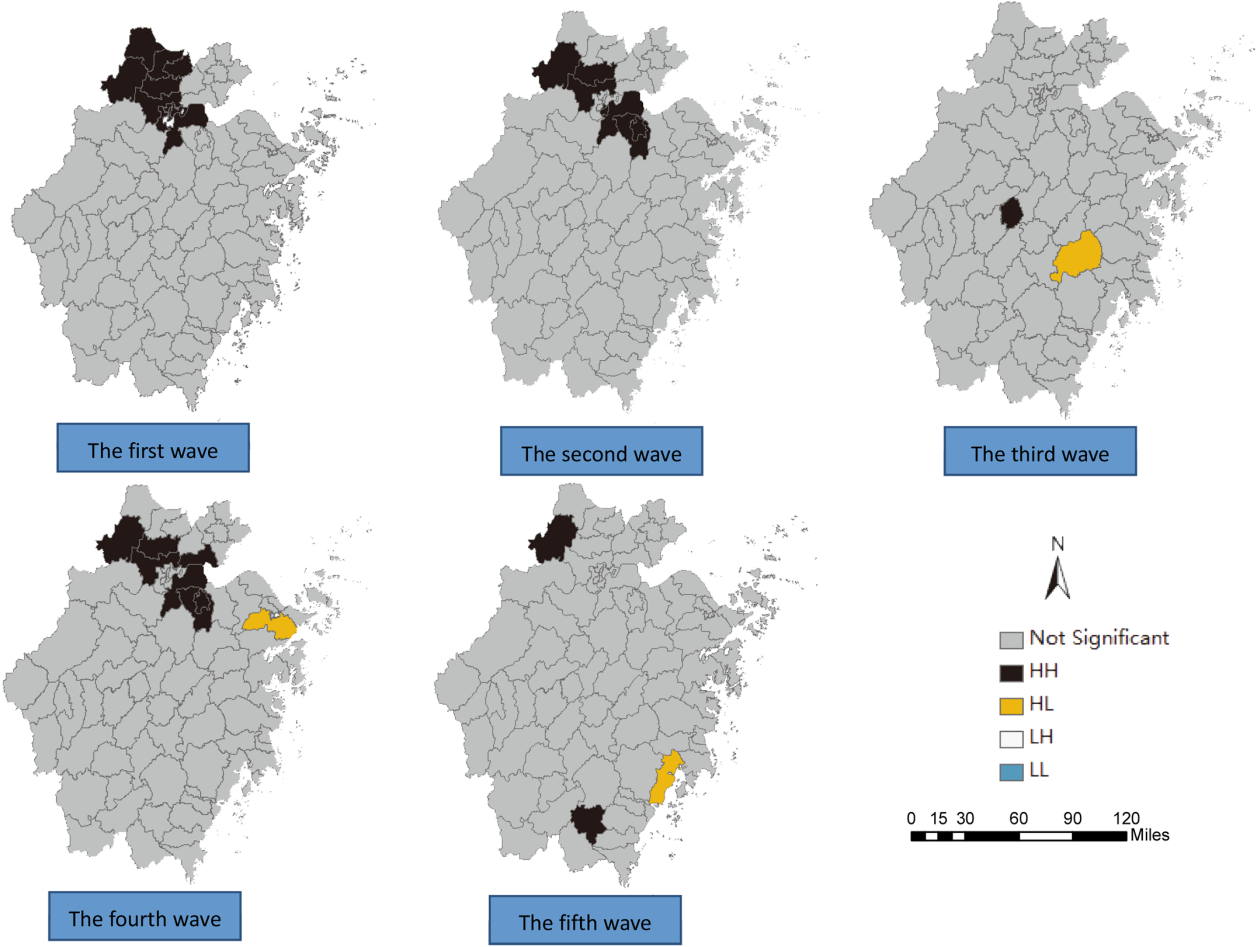
Maps of the local autocorrelation analysis of H7N9 human infections in Zhejiang Province among five waves by Local Moran’I.

These counties were all located in the northern areas of Zhejiang Province (Fig 3). There were 29 counties in this region, which accounted for 85.3% of all the areas with high-high or high-low clusters located in the northern areas of Zhejiang Province. What is interesting is that the high clusters emerged in the southern areas (Wencheng and Yueqing county) of Zhejiang Province for the first time in the fifth wave. It should also be noted that the area of the counties varied in size, with the largest being 4452 square kilometers and the smallest being 18.17 square kilometers.

### The geostatistical parameters for interpolation

**Fig 4:**
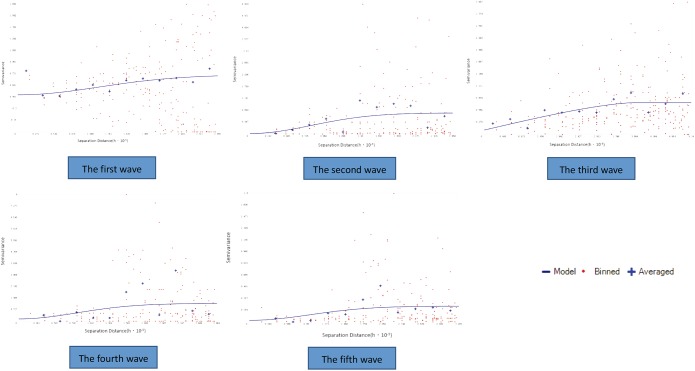
The figure of semivariogram of H7N9 human infections in Zhejiang Province among five waves.

It was shown in the semivariogram figure that the value of the semivariation function was relatively low over a short range, gradually increased as the distance between the sample points grew and then fluctuated around an extremum when the distance grew to a particular value. Similar semivariogram characteristics were shown among the five waves (Fig 4).

The partial sill (C_1_) was 0.2582, 7.7863, 0.6320, 0.9723, and 1.9801 in each wave, respectively. The nugget (C_0_) was 0.4789, 0.1642, 0.0982, 0.1416 and 0.2161 in each corresponding wave, respectively. The C_1_/ C_0_+ C_1_ ratios among the last four waves were all above 85%, and were even above 90% in the second and fifth waves, but was relatively low (35%) in the first wave, which suggested that the spatial autocorrelation was generally relatively strong. All the results are shown in Table 4.

**Table 4:** The geostatistical parameters of semivariance.

Wave	Nugget(C_0_)	Partial sill(C_1_)	Sill(C_0_+ C_1_)	Ratio(C_1_/ C_0_+ C_1_)
The first	0.4789	0.2582	0.7371	0.3503
The second	0.1642	7.7863	7.9505	0.9793
The third	0.0982	0.6320	0.7302	0.8655
The fourth	0.1416	0.9723	1.1139	0.8729
The fifth	0.2161	1.9801	2.1962	0.9016

### Estimation of the extent

**Fig 5:**
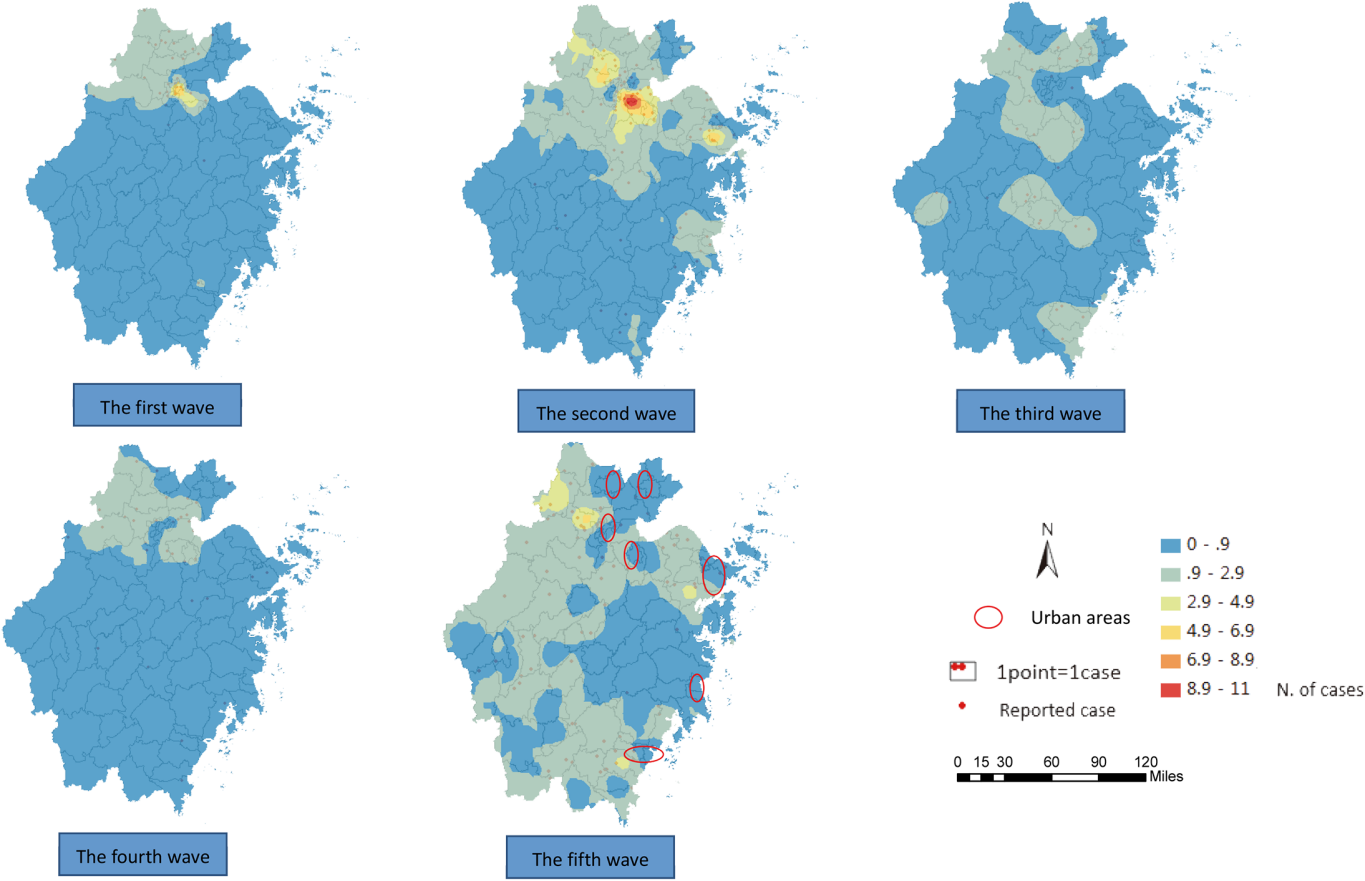
Maps of the estimation of the H7N9 human infections in Zhejiang Province among five waves epidemics by ordinary kriging interpolation.

**Fig 6:**
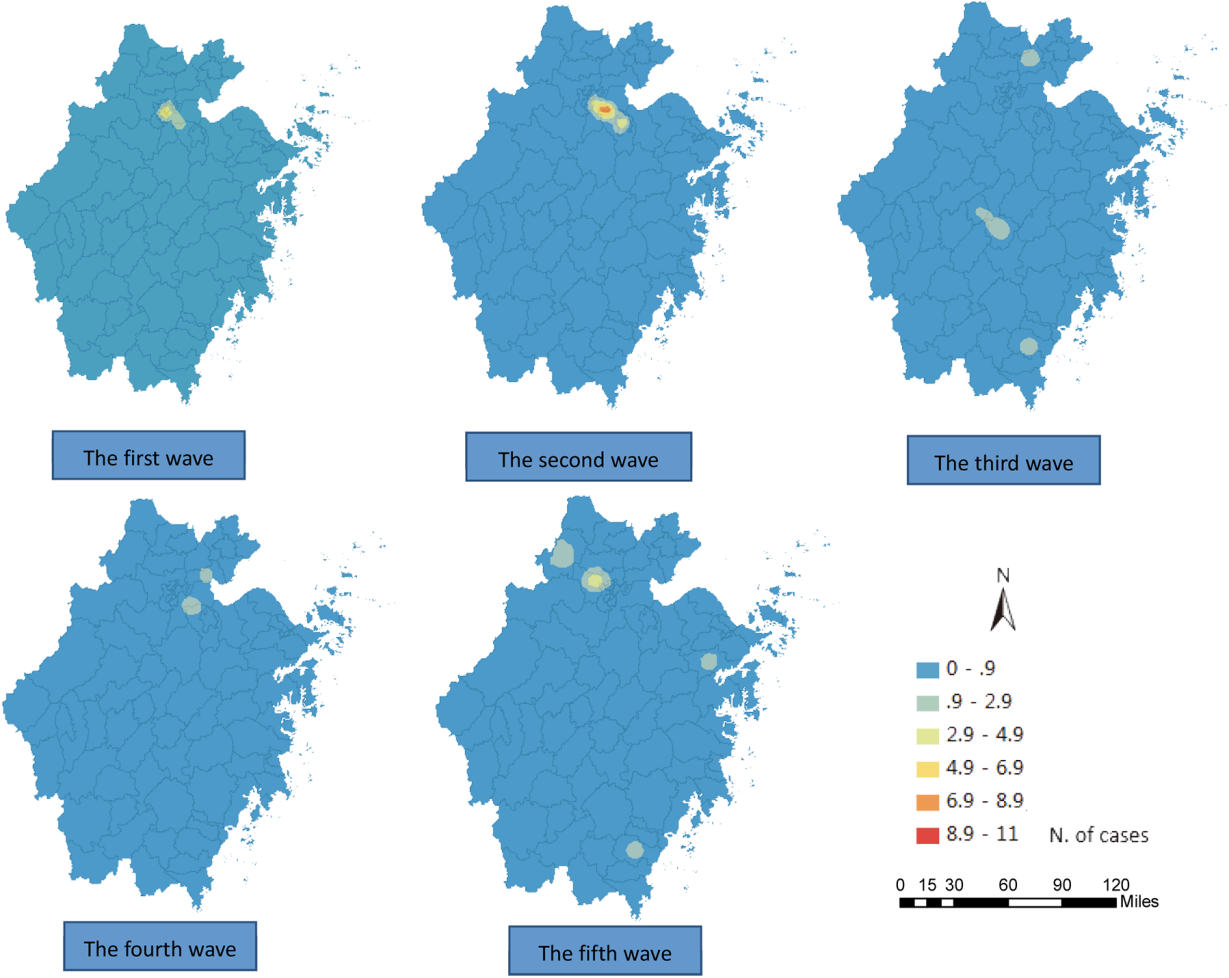
Maps of the lower-bound of confidence interval(*α* = 0.05) of the H7N9 human infections in Zhejiang Province among five waves epidemics by ordinary kriging interpolation.

**Fig 7:**
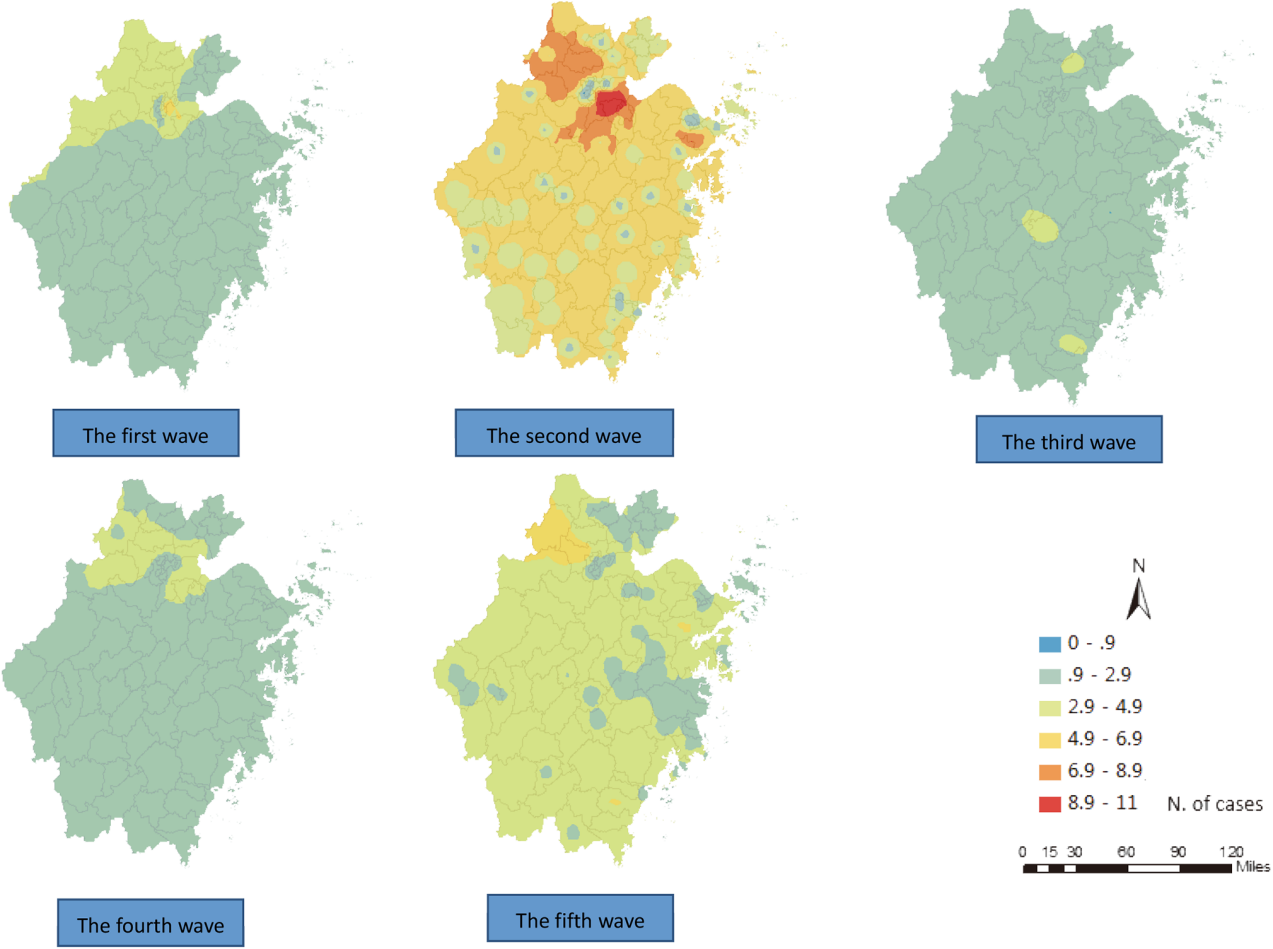
Maps of the upper-bound of confidence interval(*α* = 0.05) of the H7N9 human infections in Zhejiang Province among five waves epidemics by ordinary kriging interpolation.

The result of the ordinary kriging interpolation showed that there were many unidentified infections in the areas with no reported cases, indicating that the total number of cases infected with the H7N9 virus were far more than what has been reported to date (Fig 5). In most of the areas, there were no more than two cases within one epidemic period, and based on the estimation of the confidence interval, the number of cases were between 0–2 in the first, the third and the fourth wave. In contrast, the cases in the second and fifth wave were more than those in the aforementioned three waves and fluctuated between 0–6 in the second wave and between 0–4 in the fifth wave in most of the areas (Figs 6 and 7).

Second, the epidemics continuously circulated in the northern areas, including Hangzhou City, Jiaxing City, Huzhou City and Shaoxing City in Zhejiang Province throughout the five waves (Figs 5–7), and spread to the southern and central areas since the second wave (Figs 5 and 7). The estimated number of cases in the northern areas were obviously more than those in the other areas, especially in the second wave, where the cases in Xiaoshan county, Yuhang county, Deqing county, Anji county and Yinzhou county were all above 7 (Fig 7). Ningbo City, Taizhou City and Wenzhou City in the southeast areas were affected in the second wave, and Jinhua City, Quzhou City and Lishui City located in the south-central areas were also affected in the waves that followed. The affected areas in the fifth wave were larger than those in previous waves. It was found that most cities in Zhejiang Province were affected by the epidemics throughout all five waves, but only Zhoushan City, located on the east island, remained virus free (Figs 5 and 6), and there were perhaps no more than two cases in Zhoushan City, according to the upper-bound of the estimation (Fig 7). It should also be noted that one highly-infected region in the east area of Ningbo City and one highly infected region in the south were identified in the second and the fifth wave, respectively.

Third, according to the estimation maps of H7N9 human infections, there was an interesting result that the cases that emerged in the urban areas declined significantly. Since the fourth wave, most cases occurred in rural areas, while in the previous three waves both urban and rural areas were influenced (Fig 5).

Lastly, the spatial aggregation enhanced stepwise relative to the previous two waves based on the definition of the cluster[14]. The epidemic presented some relatively dispersed characteristics since the third wave with cases reported fewer than two in most counties, but the epidemic showed a reinforcing trend again in the fifth wave (Figs 5 and 7). In general, the affected areas continued to expand, and the epidemic may have been circulated in some new areas with no previously reported cases (Figs 5 and 7).

## Discussion

Similar to previous research [3,10], most of the cases infected with H7N9 virus were males and people over 50 years old, and there was a higher proportion of farmers. The epidemic characteristics, including gender, age and occupational distribution, were similar across the five waves in the Zhejiang Province in China. Although there were significant differences in death rate, the fatality rate was similar among the last four waves and there was no significant trend in death rate across the five waves. The reason for these similarities in the epidemic characteristics among the five waves may be because the circulating H7N9 virus genotypes were similar to those in previous waves, although there were a few point mutations discovered in the amino acids in some samples, which might increase the virus’ adaptation to gain person-to-person transmission ability and infect humans more efficiently [21–23]. The conclusion to date was that the genetic changes have not been sufficient enough to alter the antigenic characteristics or cause sustained human-to-human transmission[8]. In the fifth wave, there were more cases occurring in December 2016 than in the corresponding periods in previous years. Its peak incidence continued in January 2017 and then fell rapidly. Meanwhile, the incidence peak may move up. The reasons for the advance of the peak could be that the temperature in December 2016 was suitable for the virus and the advance of the Chinese New Year in 2017, which increased live poultry consumption and exposure[24].

The key assumption of the interpolation analysis was that the distribution of the value was a spatial correlative[19,34]. As we know, most of the infectious diseases were spatially correlative depending on the geographical environment, meteorological factors, economic levels, local custom and other factors. The H7N9 epidemic emerged in the Northern areas of the Zhejiang Province, then the spread of the virus and the expansion of the contaminated region contributed to the dispersion of the epidemic. The similarity of the contaminated environment and the exposure to the virus make the case spatially correlative and the interpolation analysis feasible.

Additionally, we used some parameter to evaluate the assumption of the model. The semivariance parameter is usually used to evaluate the strength of the correlation, except for Moran's I Index. According to the semivariogram figure, the semivariance was relatively low in the short range and gradually grew to the extremum among the five waves. This means that the more close sample points, the characteristics of the sampleswere more similar. The ratio of the partial sill to the sill was the key indicator, which represents the extent of variation due to spatial autocorrelation. In the study, these ratios among the last four waves were all above 85%, which suggested that the spatial autocorrelation was relatively strong in general and the variation due to the spatial autocorrelation was the main contribution to the observed difference. The ratio in the first wave was relatively low(35%), the reason of which maybe the cases in the first wave almost clustered in the northern areas and the different epidemics in the northern areas relative to other areas, however the local spatial autocorrelation in the northern areas was still strong. Therefore, the interpolation analysis was suitable for the incidence of H7N9 infection among the five waves.

Ordinary kriging interpolation was widely used to estimate the extent and geographical distribution for infectious diseases such as HIV, West Nile Disease and Dengue fever [25–27]. Kriging is an effective supplement for regular case surveillance, which depends on the medical personnel report. According to the inference of some previous research, there were mild and subclinical infections occurring at the same time as the epidemic. In addition to the severe cases, the number of cases of symptomatic H7N9 virus infections may be approximately 500–1000 times that of the laboratory-confirmed cases [28–30]. Consistent with the previous research, it was found that areas with possible unidentified cases were larger. The number of cases could be more than those reported to date and most of them may be the mild or subclinical ones.

The epidemic is continuously circulating in the northern areas in Zhejiang Province, China throughout the five waves. Most of the long-term high-high clusters were identified in the northern areas, which meant the incidences of h7n9 infection were similarly high among these areas, and more attention is needed to prevent epidemics in the areas. The possible reason for the continuous epidemic and clusters in the northern area is that these areas are in the Yangtze River Delta region along the Taihu Lake, which is recognized as the origin of virus recombination that lead to the outbreaks [9,31]. Therefore, the circulation of the virus in the domestic poultry transmitted by the local wildfowl and contaminated environment sustained, which contributes to the enzootic H7N9 virus infection in such areas. Although permanent closure of the live poultry markets was implemented in the main urban areas of Zhejiang Province, live poultry trade in rural areas and in privacy or wholesale crossing the border still existed. This contributed to the spread of the virus from the northern contaminated region to other counties and the expansion of the affected areas, especially the southern and central areas of Zhejiang Province [9,31]. Based on our study, Wenzhou city, located in the southeast area of Zhejiang, possibly became another subsource of H7N9 outbreaks. One high-high cluster was found in the Wencheng county in the fifth wave, which meant similar epidemics in this county and its neighboring areas. Additionally, 3 high-low clusters were found in the southeast areas. Although incidences in their surrounding areas were lower, the epidemic may expand to the neighboring areas where it may become a source of H7N9 outbreak in the future. One important thing that should be noted is that the continuous trans-regional expansion of the H7N9 viruses has accelerated the reassortment among H7N9 and other subtypes of viruses [9], which would make the viruses more mammal-adapted as well as allow them to become human-to-human transmissible and lead a pandemic in the future [21].

It was proven that the closure of the live poultry markets (LPM), which were the main place of live poultry transaction in urban areas, was highly effective in reducing the risk of H7N9 infection in humans [32]. In our study from the estimation maps, it was found that the epidemics tended to disappear in urban areas and presented relatively sporadic characteristics, especially since the fourth wave after the closure of the LPM in the urban areas in 2014. Meanwhile, according to the total proportion of rural cases, it increased obviously in the fourth and the fifth waves. However, different from urban areas in Zhejiang Province, live poultry transactions in rural areas were mainly scattered among the whole area and not centralized in markets. For example, many farmers sell poultry at the roadside instead of in the market. This may be one reason for the increasing proportion of rural cases. According to the latest research [33], raising backyard poultry at home and having direct contact with backyard poultry have been identified as two new risk factors associated with increased risk of infection with H7N9 virus, which may be another reason that the epidemic currently circulated primarily in the rural areas in the Zhejiang Province instead of in urban areas.

Since the third wave, the affected areas expanded and the rural cases increased abruptly, especially in the fifth wave, although the cases were fewer than two in most of the rural areas. However, because of the active live poultry trading and the custom of raising backyard poultry, the virus is very easily transmitted between the village, which leads to the expansion of the contaminated environment and the circulation of the epidemic in new areas. This may increase the risk of exposure to the virus and the possibility of a potential cluster. Additionally, this maybe the reason for the reinforcing trend in the fifth wave.

Several limitations should be noted with our study. First, data, including the clinic information, environmental specimens and serological surveillance, were not collected, and thus detailed clinical characteristics, positive rates of H7N9 virus in the environment and seropositive rates could not be compared. Second, only those cases confirmed to be infected with the H7N9 virus were analyzed by the ordinary kriging interpolation methods. If the seropositive rate of the population and the positive rate of H7N9 in the environment were added into the analysis, the estimation would have been more accurate. Finally, the extent of infection was estimated only in the spatial as opposed to the temporal dimension. Therefore, the temporal trend of the epidemic in each area could not be identified. Further research should be carried out to obtain a more comprehensive conclusion.

## Conclusion

In conclusion, this study revealed that there were few differences in the epidemiologic characteristics among the five waves of the H7N9 infection epidemics. The epidemic was relatively dispersed, but areas with a possible circulating epidemic were larger than what has been reported, especially in the fifth wave. The epidemic mostly occurred in rural areas, and cross-regional expansion continued since the fourth wave. Based on the findings, we suggest continuous closure of the LPM in both rural and urban areas. Illegal and scattered live poultry trading, especially in rural areas, must be forbidden. In addition, we should strengthen health education about self-protection from feeding backyard poultry and slaughtering poultry by hand. Moreover, the management of the live poultry trade and wholesale border crossing should be more rigorous to prevent the spread of the virus across the area. The surveillance of the cases, especially the mild ones, should be more heightened. Lastly, ongoing research of the pathogenicity in the internal genes is needed.

## Supporting information

S1 File The data base of key information of the case infected with H7N9 virus among five waves in Zhejiang Province. (XLS)

S2 File The certification of the manuscript was edited for proper English language. (PDF)
